# Enhanced diabetes prediction using CTGAN-MLP approach on body composition data

**DOI:** 10.1038/s41598-025-31928-9

**Published:** 2025-12-10

**Authors:** Javad Hassannataj Joloudari, Mohammad Maftoun, Mohammad Ali Nematollahi, Kandala N. V. P. S. Rajesh, S. Prasanth Vaidya, Kamireddy Rasool Reddy, Pirhossein Kolivand

**Affiliations:** 1 Research Center for Health Management in Mass Gathering, Red Crescent Society of the Islamic Republic of Iran, Tehran, Iran; 2 Department of Computer Engineering, Bab.C, Islamic Azad University, Babol, Iran; 3 Department of Computer Engineering, Faculty of Engineering, University of Birjand, Birjand, Iran; 4 Department of Computer Engineering, Technical and Vocational University (TVU), Tehran, Iran; 5 Department of Artificial Intelligence, Technical and Engineering Faculty, South Tehran Branch, Islamic Azad University, Tehran, Iran; 6 Department of Computer Sciences, Fasa University, Fasa, Iran; 7 School of Electronics Engineering, VIT-AP University, Vijayawada, 522241 AP India; 8 Department of CSE, BV RIT HYDERABAD College of Engineering for Women, Hyderabad, Telangana India; 9 Department of Electronics and Communication Engineering, St. Martin’s Engineering College, Dhulapally, Secunderabad, 500100 Telangana India; 10 Department of Health Economics, Faculty of Medicine, Shahed University, Tehran, Iran; 11 Research Center for Emergency and Disaster Resilience, Red Crescent Society of the Islamic Republic of Iran, Tehran, Iran

**Keywords:** Diabetes prediction, Body composition data, Generative adversarial networks, Machine learning, Deep learning, Multilayer perceptron, Computational biology and bioinformatics, Diseases, Health care, Mathematics and computing, Medical research

## Abstract

Accurate diabetes risk prediction is essential for timely intervention and effective disease management. To address these issues, this study evaluates a prediction framework that incorporates Conditional Tabular Generative Adversarial Network (CTGAN) to generate additional synthetic samples and mitigate class imbalance. Unlike interpolation-based oversampling techniques, CTGAN models the underlying data distribution and may better preserve nonlinear relationships among body composition variables. When combined with a Multilayer Perceptron (MLP), this approach enables the model to capture complex feature interactions that could be relevant for distinguishing individuals with diabetes from healthy participants. In our experiments, the CTGAN-augmented MLP achieved an accuracy of 93.91%, an AUC of 93.87%, a precision of 94.48%, and an F1-score of 93.89% under stratified 5-fold cross-validation, representing the highest performance among the evaluated models. The SHapley Additive exPlanations (SHAP) analysis was further employed to enhance interpretability and provided insight into the contribution of key predictors such as fat percentage, fat-free mass, and basal metabolic rate.

**Supplementary Information:**

The online version contains supplementary material available at 10.1038/s41598-025-31928-9.

## Introduction

Diabetes mellitus is a chronic metabolic disease characterized by dysregulated blood glucose levels resulting from inadequate insulin production or reduced insulin sensitivity^1^. It continues to pose a growing global health challenge, fueled by sedentary lifestyles, poor dietary habits, and genetic predisposition^2^. Clinically, diabetes is classified into three main types: Type 1 Diabetes (T1D), Type 2 Diabetes (T2D), and Gestational Diabetes (GD). T1D is an autoimmune condition in which pancreatic β-cells are destroyed, typically presenting during childhood or adolescence^3^. T2D, which accounts for more than 90% of all cases, is characterized by insulin resistance combined with impaired insulin secretion and is more prevalent in adults^4^. GD emerges during pregnancy and usually resolves after delivery, but significantly increases the mother’s risk of developing T2D later in life^5^.

Although symptoms vary, common manifestations include excessive thirst, frequent urination, unexplained weight loss, blurred vision, and delayed wound healing^6^. According to the International Diabetes Federation (IDF) and the World Health Organization (WHO), an estimated 537 million adults aged 20–79 were living with diabetes in 2021. This figure projected to increase to 783 million by 2045^7^. In the same year, diabetes was responsible for 6.7 million deaths worldwide and generated healthcare costs exceeding USD 966 billion^8^. Alarmingly, nearly 45% of affected individuals remain undiagnosed, resulting in preventable complications and a growing economic burden on healthcare systems^9^.

Early and accurate diagnosis is therefore essential to prevent life-threatening complications such as cardiovascular disease, nephropathy, neuropathy, and retinopathy. Conventional diagnostic methods, including fasting blood glucose and oral glucose tolerance tests, are effective. However, they are time-consuming, invasive, and subject to biological variability^10^. Recent advances in Artificial Intelligence (AI)-aided decision-making technologies, such as Machine Learning (ML) and Deep Learning (DL), have opened new possibilities for diabetes risk prediction by uncovering hidden patterns in complex clinical data^11–13^. However, the performance of these models is often hindered by real-world challenges such as missing values, class imbalance, and overfitting, which limit their generalizability^14^.

To address these issues, this study evaluates a prediction framework that incorporates Conditional Tabular Generative Adversarial Network (CTGAN) to generate additional synthetic samples and mitigate class imbalance. Unlike interpolation-based oversampling methods, CTGAN models the underlying data distribution and may better preserve nonlinear relationships among body composition variables. When combined with a Multilayer Perceptron (MLP), this approach allows exploration of complex feature interactions that could be relevant for identifying individuals at higher risk of diabetes. The SHapley Additive exPlanations (SHAP) analysis is further conducted to improve interpretability and provide insights into the contribution of individual predictors.

The main contributions of this work are summarized as follows:

1. Real-World Cohort Data: We employed a subset of the Iranian Fasa Cohort Study, which contains detailed body composition measurements and presents real-world issues such as outliers, missing values, and severe class imbalance.
2. Advanced Data Augmentation: By using CTGAN to generate realistic and physiologically consistent synthetic data that maintain the complex correlations among body composition features, CTGAN alleviated class imbalance in contrast to Synthetic Minority Oversampling Technique (SMOTE) and Adaptive Synthetic Sampling (ADASYN), preserved the statistical integrity of metabolic variables, and thus improved the robustness and generalizability of the models.
3. Superior Predictive Performance: The CTGAN–MLP framework effectively captured nonlinear physiological interactions within body composition data. Leveraging CTGAN-generated samples, the MLP achieved 93.91% accuracy and the highest overall performance across area under the curve (AUC), F1-score, Matthews correlation coefficient (MCC), and geometric mean (G-Mean), demonstrating strong statistical reliability and clinical significance.
4. Explainable AI: By integrating advanced augmentation with an interpretable MLP model and SHAP analysis, this framework provides a scalable decision-support tool for early diabetes detection and personalized risk assessment.

The remainder of this paper is organized as follows: Sect. “Related work” reviews related work on diabetes prediction. Section “Traditional ML methods” describes the materials and methods. Section Ensemble and hybrid ML methods presents experimental results, and Sect. “Deep learning methods” provides the limitations of this study. Finally, Sect. “Materials and methods” discusses key findings and future research directions.

## Related work

Diabetes remains a major global health challenge, driving a growing interest in predictive modeling and risk assessment. To address this, researchers have applied a wide spectrum of data-driven approaches, ranging from classical ML algorithms to advanced DL and AI methods. These studies aim not only to estimate prevalence more accurately but also to enable earlier and more reliable prediction of disease onset.

To provide a structured overview, this section is organized into three parts. Section “Traditional ML methods” reviews traditional machine learning approaches commonly used for diabetes prediction. Section “Ensemble and hybrid ML methods” focuses on ensemble and hybrid methods that combine multiple algorithms for improved performance. Finally, Sect. “Deep learning methods” highlights recent advances in deep learning techniques applied to diabetes prediction.

### Traditional ML methods

Guariguata et al. employed Logistic Regression (LR) on data from 565 sources stored in a MySQL database, highlighting advantages such as simplicity, adaptability, and reproducibility^15^. However, their method lacked age-specific estimates and did not consider lifestyle or obesity factors. Peña et al. incorporated anthropometric and biochemical measurements, physical activity, and diet to estimate type 2 diabetes prevalence in Mexico City, accounting for crucial lifestyle factors but unable to establish strong diet–exercise relationships due to sample limitations^16^. Lee and Kim investigated T2D risk in Korean adults using anthropometry, Waist Circumference (WC), and triglycerides (TG) with binary LR and Naive Bayes (NB) and 10-fold cross-validation; however, reliance on raw WC and TG values limited generalizability and causal interpretation^17^. Zhu et al. examined abdominal fat distribution using CT imaging and LR but did not analyze fat distribution among healthy individuals^18^.

### Ensemble and hybrid ML methods

Elgendy et al. introduced an explainable two-stage ensemble combining Local Outlier Factor (LOF), autoencoders,

SMOTE, and SHAP on the MIMIC-IV dataset, achieving 92.54% accuracy^19^. Additionally, they proposed a graph-based framework modeling relationships among patients with similar conditions through a patient network, employing centrality measures and demographic features to train multiple classifiers. The Random Forest model performed best, with the AUC values between 0.79 and 0.91, demonstrating the value of latent structural information in prediction. Building on this, in^20^, a classification pipeline was developed using a weighted ensemble of five machine learning models, including Decision Tree (DT), Random Forest (RF), Extreme Gradient Boosting (XGBoost), and Light Gradient Boosting Machines (LightGBM). Key preprocessing steps involved missing value imputation, feature selection, and hyperparameter tuning via grid search. This ensemble achieved an accuracy of 73.5% and an AUC of 0.832, showing substantial performance improvement over individual models.

Naseem et al. implemented a patient health monitoring system utilizing six machine learning models, including both traditional and deep learning approaches^21^. Among them, the Recurrent Neural Network (RNN) achieved the highest accuracy (81%), while the Artificial Neural Network (ANN) delivered the highest recall. This system was designed to assist early diagnosis of chronic diseases by leveraging ML-based decision support. Nematollahi et al. further explored diabetes prediction on Fasa cohort data, applying XGBoost with oversampling via ADASYN to address class imbalance, achieving 89.96% accuracy^22^. Moreover, a notable recent study by Nematollahi et al. examined the association between body fat distribution and diabetes using machine learning and Analysis of Variance (ANOVA)^14^. By combining individual classifiers and ensemble learning, alongside ADASYN oversampling, they reached an impressive 92.04% accuracy using XGBoost.

To improve classification approaches, authors in^23^ proposed a hybrid Support Vector Machine (SVM) kernel based on Radial Basis Function (RBF) and city-block metrics. They addressed class imbalance with SMOTE and data quality via median imputation. The model achieved 87% precision, underscoring its potential for clinical diagnostics of T2D. In^24^, an ensemble-based AI system was developed employing Harmony Search for feature selection and hyperparameter optimization. Tested on both Western and Eastern medical datasets, the system attained 93.09% accuracy on the PIMA dataset, demonstrating efficient model complexity reduction while maintaining strong predictive power.

### Deep learning methods

The knowledge Extension with Convolution Neural Network (KE-CNN) model introduced in^25^ applied a hybrid deep learning approach combining medical entity recognition with semantic knowledge expansion. Utilizing tools such as Bidirectional Encoder Representations from Transformers-Bidirectional Long Short-Term Memory-Conditional Random Fields (BERT-BiLSTM-CRF) and Word2Vec, this model captured richer feature representations, improving diabetes prediction. Its dual-channel CNN framework further enhanced accuracy by integrating structured and unstructured data inputs.

Furthermore, Mushtaq et al. proposed a voting ensemble classifier composed of Naive Bayes (NB), Random Forest (RF), and Gradient Boosting (GB) models to mitigate outliers and class imbalance in diabetes datasets^26^. Data preprocessing included Tomek links for cleaning, SMOTE for balancing, and Interquartile Range (IQR)-based outlier removal. The ensemble achieved up to 82% accuracy, indicating reliability for early-stage diabetes detection. Nurzari et al. applied modified SMOTE and Random Forest classifiers, reaching an outstanding 99.7% accuracy^27^. However, despite the high accuracy reported in studies using SMOTE^27,28^, these methods suffer from limited sample diversity and issues near class boundaries.

By analyzing the reviewed approaches, it becomes clear that studies addressing class imbalance often achieve higher accuracy. However, most of these works rely on traditional balancing methods such as the SMOTE and Adaptive Synthetic Sampling (ADASYN). These methods typically generate synthetic samples by performing linear interpolation between minority class instances, which may reduce variability and introduce noisy samples near class boundaries.

In contrast, the current study explores Conditional Tabular Generative Adversarial Networks (CTGAN)^29^ for data augmentation. Unlike SMOTE, ADASYN, and CTGAN learns the full joint distribution of the dataset and generate entirely new, realistic synthetic records while preserving complex nonlinear relationships among features. This approach not only enhances the diversity of synthetic data but also reduces the risk of overfitting when training predictive models.

Moreover, while most existing studies on diabetes prediction focused on a single classifier, we conducted a systematic comparison across multiple machine learning and deep learning models to identify the most effective predictor for type 2 diabetes. This methodological enhancement, combined with CTGAN-based augmentation, represents the key novelty of our research within the context of the Fasa cohort.

## Materials and methods

In this study, we present a structured and interpretable framework for predicting diabetes using body composition data. The pipeline begins with Data Source and Ethical Considerations, relying on the ethically approved Fasa Cohort Study. Next, Data Preprocessing is performed to address missing values, categorical encoding, and potential outliers, followed by Feature Scaling through MinMaxScaler to ensure standardized inputs. To mitigate class imbalance, Data Augmentation methods such as CTGAN, SVM-SMOTE, RandomUnderSampler, and ADASYN are applied. The dataset is then partitioned through Data Splitting and Cross-Validation (stratified 5-fold), and a broad range of algorithms are explored under the Modeling stage, including machine learning and deep learning approaches. Each model undergoes Hyperparameter Tuning to optimize predictive performance, and outcomes are systematically assessed under Model Evaluation using diverse metrics. Finally, Result Interpretation is performed to identify the best-performing approach and highlight clinically meaningful predictors.

**Fig. 1:**
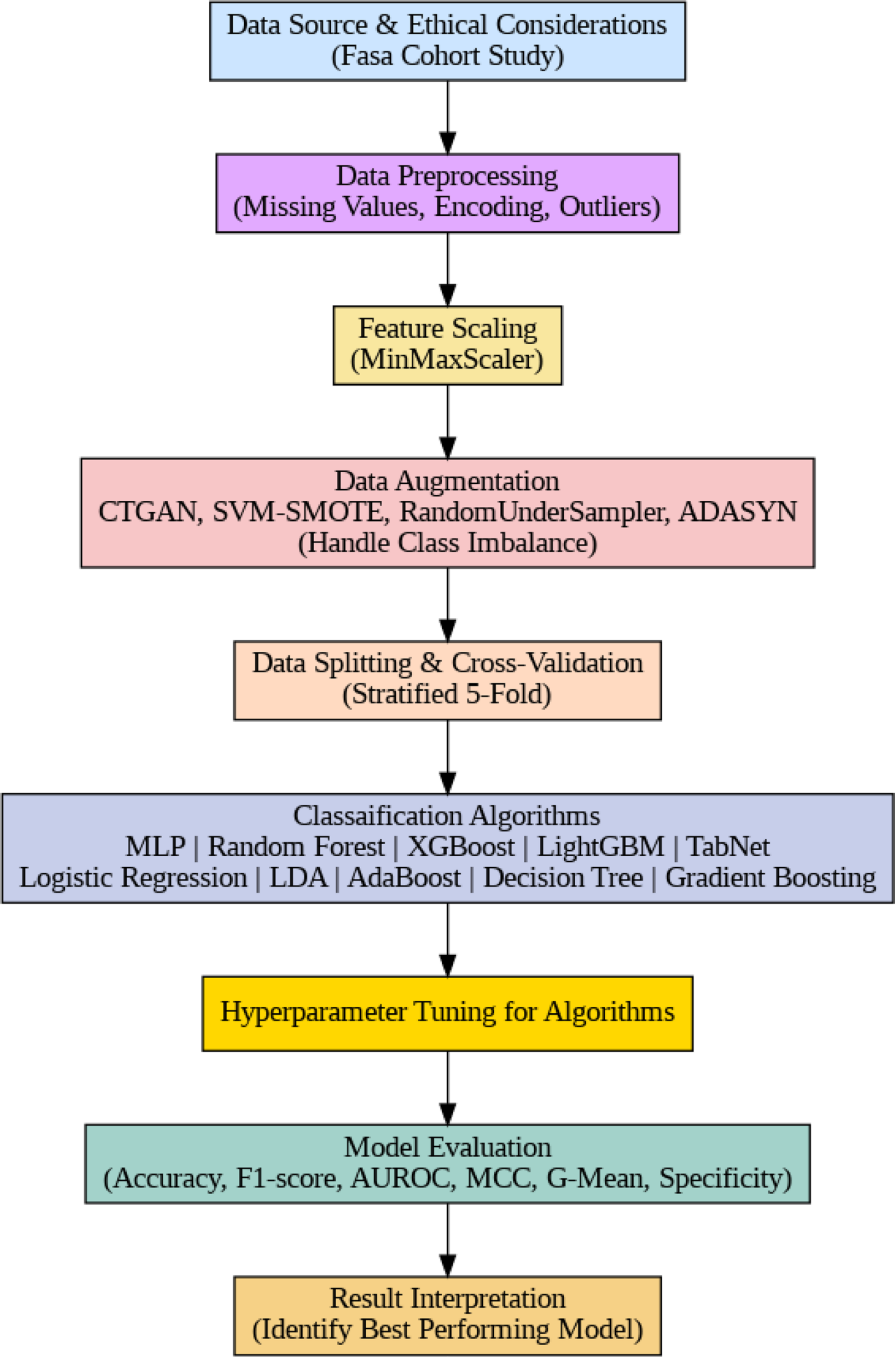
Block diagram of the proposed methodology.

### Data source and ethical considerations

The dataset used in this study is a subset of the Fasa Cohort Study, conducted in Iran^30^, which investigates associations between risk factors and chronic non-communicable diseases among rural residents of Fasa, Fars Province^22^. This subset focuses on participants with diabetes and healthy individuals, incorporating their body composition measures. It includes 4,661 participants (2,155 males, 2,506 females), aged between 35 and 70 years. Participants were included if they were within this age range and either had a diagnosis of diabetes or were considered healthy based on clinical assessments. Individuals with severe comorbidities, such as cardiovascular diseases, cancer, or renal failure, were excluded to minimize potential confounding effects on body composition measures. Ethical approval for this study was obtained from the Medical Ethics Committee of Shiraz University of Medical Sciences (IR.SUMS.MED.REC.1401.167), and written informed consent was acquired from all participants prior to data collection, ensuring compliance with ethical standards for research involving human subjects.

### Data preprocessing

The dataset was first examined for missing values, inconsistencies, and outliers. Missing numerical values (e.g., Age) were imputed using the mean, while missing categorical values (e.g., GenderID, HasDiabetes) were imputed using the mode. To ensure compatibility with the machine learning pipeline, categorical features were converted into a numeric format through encoding techniques. Formally, this transformation can be expressed as:

1 $$\:X\_encoded\:=\:OneHotEncode\left(X\right)$$

where $\:X\_encoded\:$represents the encoded feature space. This preprocessing step facilitated the uniform representation of heterogeneous variables and reduced bias arising from categorical data handling.

### Feature scaling

To bring all numerical variables into a comparable range and improve model convergence, MinMax scaling was applied. This technique rescales each feature to the interval$\:\:\left[\mathrm{0,1}\right]$ while preserving the original distribution. The transformation can be formally defined as:

2 $$\:X\_scaled\:=\:X\_scaled\:=\:(X\:-\:X\_min)\:/\:(X\_max\:-\:X\_min)\:\:\:\:\:$$

where $\:X\_min$ and $\:X\_max$ denote the minimum and maximum values of the feature, respectively. This step ensured that features with different scales contributed equally during model training.

### Data augmentation

To mitigate class imbalance, we employed a hybrid of generative and resampling methods, explicitly changing only a few hyperparameters while letting all other settings remain at their library defaults. We Initialized CTGAN via CTGAN () (i.e., using default settings for embedding, network architecture, learning rate, etc.), and only modified the number of epochs to 200 to produce synthetic minority-class samples until balance was achieved. Alongside that, we applied SVM-SMOTE (SVM-SMOTE (), defaults: sampling_strategy=’auto’, k_neighbors = 5, m_neighbors = 10, etc.), RandomUnderSampler (RandomUnderSampler (), default sampling_strategy=’auto’)^31^, and ADASYN (ADASYN (), defaults such as sampling_strategy=’auto’, n_neighbors = 5) to resample^32^. SVM-SMOTE focuses on generating new minority samples near decision boundaries, RandomUnderSampler randomly removes majority samples, and ADASYN adaptively generates more samples in sparsely populated regions, thereby enhancing the classifier’s ability to learn from difficult cases^33^.

### Data splitting and Cross-Validation

We adopted stratified K-Fold cross-validation (k = 5) to ensure proportional representation of each class in all folds. This procedure improved the robustness and generalizability of the results.

### Classification algorithms

In this study, we considered a diverse set of machine learning algorithms to ensure comprehensive evaluation across different model families. The selected models included Logistic Regression (LR), Decision Tree (DT), Random Forest (RF), AdaBoost, GB, LightGBM, XGBoost, MLP, and TabNet. This selection was motivated by their frequent use in medical informatics, their ability to handle heterogeneous feature spaces, and the balance they provide between interpretability and predictive power.

Classical linear methods such as LR and Linear Discriminant Analysis (LDA) have long been considered reference baselines in clinical prediction tasks. It is due to their statistical interpretability and straightforward implementation^34,35^. Tree-based learners, including DT, RF, and boosting methods, including AdaBoost, GB, LightGBM, and XGBoost, were incorporated for their robustness against noise, ability to model nonlinear relationships, and consistent success in disease prediction contexts^36–38^. Neural-based approaches such as MLP extend the capacity to capture higher-order interactions among features, though at the cost of reduced interpretability [40]. Finally, TabNet was included as a recent deep learning model tailored for tabular data; it employs sequential attention mechanisms to achieve both competitive performance and feature-level interpretability, which is of particular value in clinical applications.

### Hyperparameter tuning for algorithms

In our study, we trained ten algorithms simultaneously, as shown in Fig. 1. Table 1 presents the hyperparameters used for all these models.

**Table 1:** Hyperparameters considered for machine learning algorithms.

Algorithms	Parameters
MLP	hidden_layer_sizes=(100,), activation=‘relu’, solver=‘adam’, alpha = 0.0001, batch_size=‘auto’, learning_rate=‘constant’, max_iter = 200
Gradient Boosting	loss=‘log loss’, learning_rate = 0.1, n_estimators = 100, max_depth = 3, min_samples_split = 2, min_samples_leaf = 1, subsample = 1.0
LightGBM	boosting_type=‘gbdt’, num_leaves = 31, learning_rate = 0.1, n_estimators = 100, max_depth=−1, min_child_samples = 20, reg_alpha = 0.0, reg_lambda = 0.0
XGBoost	booster=‘gbtree’, learning_rate = 0.3, n_estimators = 100, max_depth = 6, min_child_weight = 1, subsample = 1.0, colsample_bytree = 1.0, reg_alpha = 0, reg_lambda = 1
Random Forest	n_estimators = 100, criterion=‘gini’, max_depth = None, min_samples_split = 2, min_samples_leaf = 1, bootstrap = True
TabNet	optimizer_fn = torch.optim.Adam, optimizer_params=‘lr’: 5e-4, scheduler_fn = torch.optim.lr_scheduler.StepLR, scheduler_params=‘step_size’:10, ‘gamma’:0.9, mask_type=‘entmax’, max_epochs = 100, patience = 100, batch_size = 256, drop_last = False
Logistic Regression	penalty=‘l2’, C = 1.0, solver=‘lbfgs’, max_iter = 100, multi_class=‘auto’
AdaBoost	n_estimators = 50, learning_rate = 1.0, algorithm=‘SAMME.R’
LDA	solver=‘svd’
Decision Tree	criterion=‘gini’, splitter=‘best’, max_depth = None, min_samples_split = 2, min_samples_leaf = 1

## Results

In this section, we present the experimental findings of our proposed diabetes prediction framework. All experiments were conducted in the Google Colab environment, using a pre-processed dataset derived from the Fasa Cohort Study. To ensure clarity, the results are organized into several subsections. Section “Body composition measures” introduces the body composition measures collected from participants, while Sect. “Model evaluation” outlines the evaluation metrics applied to assess predictive performance. Section “Simulation results” presents the simulation results, covering preprocessing steps, approaches to address class imbalance, and statistical feature analysis. Section “Cross-validation” explains the stratified cross-validation strategy used for model training and evaluation.

To deepen the analysis, we introduce two additional subsections. Section “Experimental performance of models” and “Real vs. synthetic data distribution of proposed method” provides simulation results and distributional comparison between real data and synthetic data generated using the proposed CTGAN-MLP approach, highlighting how well the synthetic samples preserve the statistical characteristics of the original dataset. Section “SHAP-based feature importance of the proposed MLP” focuses on model interpretability by presenting SHAP-based feature importance for the diabetes prediction model, offering insights into the contribution of individual body composition features.

Finally, Sect. “Analysis on the fusion of CTGAN-MLP” compares the proposed framework against state-of-the-art methods, enabling a comprehensive evaluation of its relative strengths and advancements.

### Body composition measures

All participants were assessed for body composition using the FDA-approved Tanita Segmental Body Composition Analyzer BC-418 MA (Tanita Corp, Japan)^22^. The data was collected while each subject stood barefoot on the device while gripping the attached handles, allowing bioelectrical impedance measurements through eight polar electrodes at the contact points. These measurements determined total body water, fat mass, fat-free mass, fat percentage for the entire body, specific regions on the left and right sides, and basal metabolic rate.

### Model evaluation

The model’s performance is evaluated using accuracy, precision, recall, F1-score, AUC, Matthews Correlation Coefficient (MCC), and Geometric Mean (G-Mean) metrics. The formulas used to calculate each evaluation measure are presented in Eqs. (3)–(9) below.

3 $$\:\:{\rm Accuracy} =\frac{(TP\:+\:TN)}{(TP\:+\:TN\:+\:FP\:+\:FN)}$$

4 $$\:Precision\:=\:\:\frac{TP}{(TP\:+\:FP)}\:\:\:\:\:\:\:\:\:\:\:\:\:\:\:\:\:\:$$

5 $${\rm Recall} =\:\frac{TP}{(TP\:+\:FN)}$$

6 $${\rm F1\: Score} =\:\frac{2\:\times\:\:Precision\:\times\:\:Recall}{Precision\:+\:Recall}$$

7 $$\:AUROC\:=\:{\int\:}_{0}^{1}TPR\:d\left(FPR\right)$$

8 $${\rm MCC}=\:\frac{\left(\right(\mathrm{T}\mathrm{P}\:\times\:\:\mathrm{T}\mathrm{N})\:-\:(\mathrm{F}\mathrm{P}\:\times\:\:\mathrm{F}\mathrm{N}\left)\right)\:}{\sqrt{\left(\right(TP+FP\left)\right(TP+FN\left)\right(TN+FP\left)\right(TN+FN\left)\right)}}$$

9 $${\rm G-Mean}=\:\sqrt{Recall\:\times\:\:Specificity}$$

Based on the definitions presented in the formulas, the key evaluation terms used in the performance metrics are explained as follows:

- **True Positives (TP)**: The number of samples that actually belong to the positive class and are correctly predicted as positive by the model.
- **True Negatives (TN)**: The number of samples that actually belong to the negative class and are correctly predicted as negative by the model.
- **False Positives (FP)**: The number of samples that actually belong to the negative class but are incorrectly predicted as positive by the model.
- **False Negatives (FN)**: The number of samples that actually belong to the positive class but are incorrectly predicted as negative by the model.
- **True Positive Rate (TPR)**: The proportion of actual positive samples that are correctly identified as positive.
- **False Positive Rate (FPR)**: The proportion of actual negative samples that are incorrectly identified as positive.

### Simulation results

As shown in Fig. 1, the dataset underwent several preprocessing steps before being used for model training. Missing values were addressed using three strategies: mode imputation for categorical features, mean imputation for numerical variables, and row deletion for cases where the target label was absent. All categorical attributes were subsequently converted into numerical form using the encoding scheme described in Sect. 3.2.

Table 2 provides a descriptive statistical summary of the dataset, highlighting variability across features, including central tendency, spread, and the presence of outliers. These statistics reveal substantial inter-individual differences in body composition, as well as localized skewness in segmental measures, underscoring the necessity of robust preprocessing methods. To further understand the importance of the body composition features in relation to the class labels, a correlation matrix with a corresponding heat map is provided in Figure S1 in the Supplementary Material.

**Table 2:** Descriptive statistics of dataset features, including data type, distribution characteristics, and outlier bounds.

Column	dtype	unique	min	max	median	Standard deviation	outliers	lower bound	upper bound
GenderID	int64	2	1	2	2.0	0.5	0	−0.5	3.5
Age	float64	37	35.0	70.0	46.0	9.36	0	15.0	79.0
bmr	int64	3703	1132	5837	2071.0	1019.19	87	3301.0	8557.0
FATP	float64	471	1.5	53.6	27.8	10.04	0	−2.2	57.8
FATM	float64	443	0.7	67.5	18.7	9.05	52	−5.65	42.75
FFM	float64	439	8.5	83.6	46.8	8.78	46	23.95	71.35
TBW	float64	401	20.9	62.4	34.3	6.43	46	17.55	52.35
IMP	int64	435	32	949	613.0	79.63	71	411.0	819.0
RLFATP	float64	490	1.5	55.5	32.9	12.93	0	−18.3	76.9
RLFATM	float64	104	0.1	12.9	3.5	1.91	12	−2.5	9.5
RLFFM	float64	101	4.7	18.4	7.9	1.65	43	3.45	12.65
LLFATP	float64	478	1.5	55.2	33.0	12.8	0	−17.65	76.35
LLFATM	float64	102	0.2	12.9	3.4	1.89	12	−2.48	9.33
LLFFM	float64	97	4.7	17.5	7.7	1.61	48	3.5	12.3
RAFATP	float64	490	2.3	63.4	25.3	11.48	0	−10.6	64.6
RAFATM	float64	41	1.0	5.3	2.4	0.56	109	0.45	4.35
RAFFM	float64	41	0.1	5.6	2.4	0.65	48	−0.75	4.35
LAFATP	float64	503	2.2	64.3	26.4	11.65	0	−10.65	65.75
LAFATM	float64	45	0.1	6.2	2.0	0.62	111	−0.6	4.6
LAFFM	float64	41	1.3	5.3	2.4	0.65	51	0.75	4.35
TRFATP	float64	439	3.0	51.0	26.9	9.18	2	−2.05	55.95
TRFATM	float64	244	1.0	10.3	4.4	1.46	27	−1.85	10.65
TRFFM	float64	242	15.7	44.9	26.2	4.37	52	15.0	38.2

### Cross-Validation

The next important step before training an ML is cross-validation. It decided how we split our data for training, validation, and testing. In this method, we have used a stratified five-fold cross-validation. In this validation, the dataset is first divided into five equal-sized subsets (folds) while ensuring that each fold maintains the same proportion of each class as the original dataset. The model is then trained five times, using four folds for training and the remaining fold for validation. This process helps reduce bias and variance, providing a more reliable estimate of model performance, especially for imbalanced datasets.

### Experimental performance of models

**Fig. 2:**
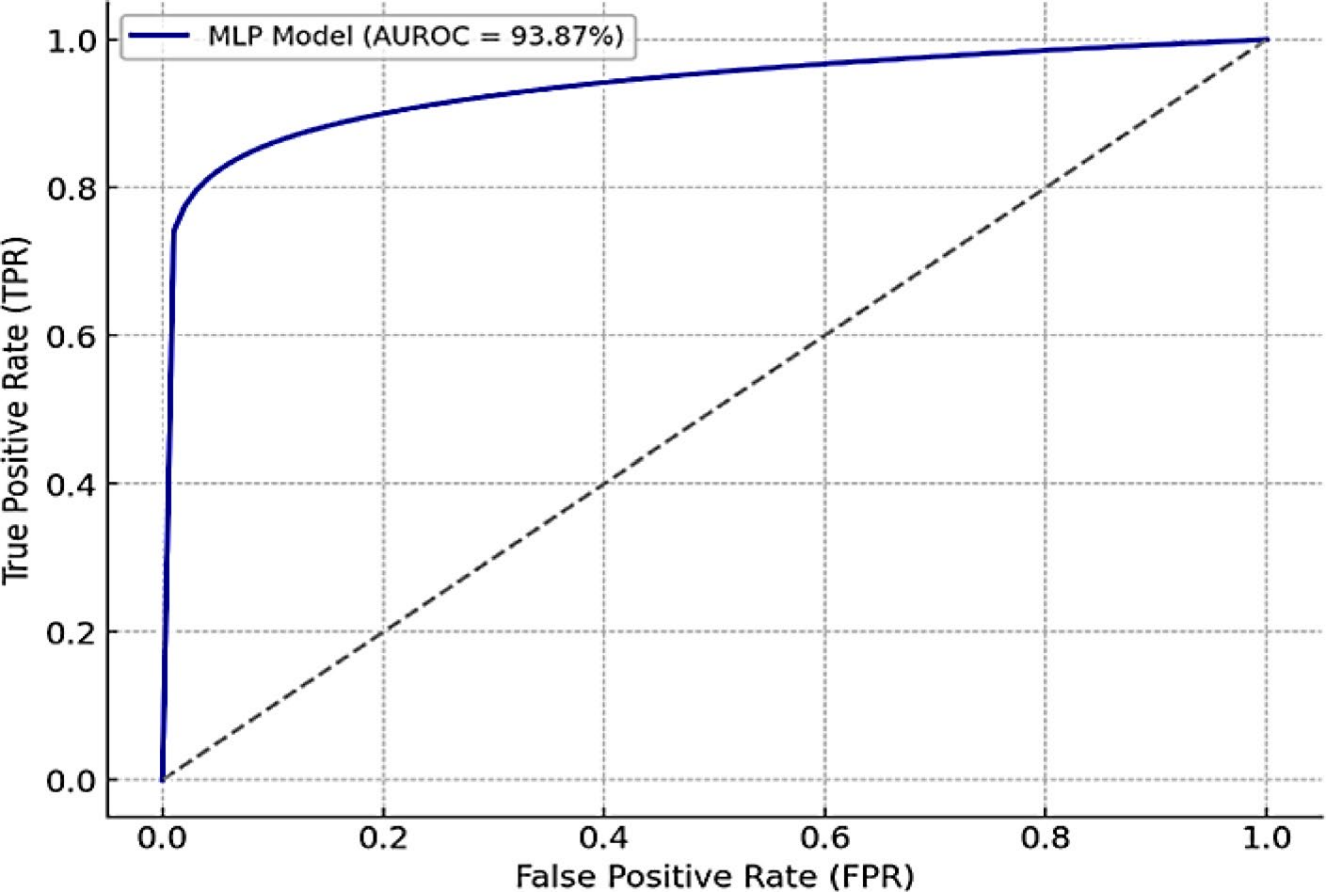
ROC curve of the best-performing MLP model.

The predictive performance of ten machine learning models was systematically evaluated under different resampling strategies, including RandomUnderSampler, SMOTE, ADASYN, and the proposed CTGAN-based approach. The detailed outcomes for each model are summarized in Tables 3, 4, 5 and 6, where the impact of various data balancing methods on model accuracy, AUROC, and other performance measures can be clearly observed. In particular, Table 3 reports the results obtained with RandomUnderSampler, Table 4 with SVM-SMOTE, Table 5 with ADASYN, and Table 6 with CTGAN-generated synthetic data. Among all configurations, the MLP classifier consistently achieved the highest predictive performance, with CTGAN-augmented data leading to the best overall results. Figure 2 further illustrates this outcome by presenting the ROC curve of the MLP model, emphasizing its strong discriminative ability.

Based on Table 3, which presents the performance of ten machine learning models using RandomUnderSampler-balanced data, overall predictive capabilities were moderate. Logistic Regression achieved the highest accuracy at 69.30%, followed closely by MLP at 68.42% and LDA at 67.54%. Boosting-based models, including Gradient Boosting and LightGBM, performed slightly lower, in the range of 63–65%. These results indicate that while under-sampling helps to address class imbalance, the reduction of majority-class samples can limit the ability of models to fully capture complex patterns in body composition data.

Based on Table 4, where SVM-SMOTE-generated synthetic data was used to augment the minority class, substantial improvements were observed across most models. XGBoost and LightGBM achieved accuracies above 92%, with corresponding geometric mean values also exceeding 92%, reflecting a better balance between sensitivity and specificity. Ensemble and boosting algorithms demonstrated the most significant gains, while models such as TabNet and LDA improved less prominently, suggesting that some architectures may be less capable of fully leveraging synthetic samples for enhanced performance.

Based on Table 5, which reports results with ADASYN-generated synthetic data, adaptive oversampling led to notable gains for ensemble models. XGBoost reached an accuracy of 91.96%, and Gradient Boosting achieved 80.61%, confirming the benefit of generating minority-class samples adaptively. However, simpler models and certain deep architectures, including TabNet, exhibited smaller improvements, indicating variability in how different algorithms exploit oversampled data.

Finally, based on Table 6, the proposed CTGAN-generated synthetic data significantly enhanced model performance across the board. The MLP model outperformed all others, achieving 93.91% accuracy, 93.87% AUROC, 94.48% precision, and 93.87% recall. Boosting models such as Gradient Boosting, LightGBM, and XGBoost consistently exceeded 92% across most metrics, while linear models remained competitive but slightly lower. These results demonstrate that CTGAN effectively preserves the statistical characteristics of the original dataset while enriching the training distribution. This augmentation enables models to capture intricate, nonlinear relationships among body composition features, particularly for deep learning architectures such as MLPs.

Because the MLP model can capture intricate, non-linear feature interactions in the CTGAN-augmented dataset, it performs better than other models. The complex, high-dimensional structures that are frequently seen in CTGAN-generated samples can make it difficult for conventional tree-based or linear models to learn. A better adaptation to the synthetic feature space is made possible by the MLP’s multilayer architecture and non-linear activation functions, which allow it to learn deeper, more abstract feature representations. It outperformed ensemble models that rely on simpler decision boundaries, as evidenced by its balanced and strong performance across several evaluation metrics, including an accuracy of 93.91% and an MCC of 88.34%.

**Table 3:** Performance metrics of ML models combined with RandomUnderSampler-balanced data.

Model	Accuracy	AUROC	Precision	Recall	F1 Score	MCC	G-Mean
Gradient Boosting	65.35	65.35	65.45	65.35	65.30	30.80	65.23
Light Gradient Boosting Machine	63.16	63.16	63.42	63.16	62.98	26.58	62.77
XGBoost	61.84	61.84	61.86	61.84	61.82	23.71	61.80
Random Forest	61.40	61.40	61.49	61.40	61.33	22.81	61.25
Tab Net	60.52	60.52	60.53	60.52	60.51	21.06	60.50
Logistic Regression	69.30	69.30	69.35	69.30	69.28	38.65	69.25
AdaBoost	65.79	65.79	66.11	65.79	65.62	31.58	65.41
LDA	67.54	67.54	67.54	67.54	67.54	35.09	67.54
Decision Tree	60.09	60.09	60.15	60.09	60.03	20.24	59.96
MLP	68.42	68.42	68.43	68.42	68.42	36.84	68.42

**Table 4:** Performance metrics of ML models combined with SVMSMOTE-generated synthetic data.

Model	Accuracy	AUC	Precision	Recall	F1-Score	MCC	G-Mean
Gradient Boosting	84.96	84.96	84.97	84.96	84.96	69.93	84.96
Light Gradient Boosting Machine	92.54	92.54	92.58	92.54	92.54	85.12	92.53
XGBoost	92.60	92.60	92.61	92.60	92.60	85.21	92.60
Random Forest	92.05	92.05	92.07	92.05	92.05	84.13	92.05
Tab Net	80.31	80.31	80.31	80.31	80.31	60.63	80.31
Logistic Regression	76.28	76.28	76.30	76.28	76.28	52.58	76.28
AdaBoost	79.10	79.10	79.12	79.10	79.09	58.21	79.08
LDA	77.87	77.87	77.90	77.87	77.87	55.75	77.86
Decision Tree	86.91	86.91	86.20	86.12	86.12	72.32	86.09
MLP	78.91	78.91	78.94	78.91	78.91	57.85	78.90

**Table 5:** Performance metrics of ML models combined with ADASYN-generated synthetic data.

Model	Accuracy	AUC	Precision	Recall	F1-Score	MCC	G-Mean
Gradient Boosting	80.61	80.55	80.75	80.55	80.56	61.30	80.47
Light Gradient Boosting Machine	92.62	92.65	92.67	92.65	92.59	85.32	92.63
XGBoost	91.96	91.97	91.96	91.97	91.96	83.91	91.96
Random Forest	91.30	91.24	91.53	91.24	91.27	82.76	91.17
Tab Net	69.14	68.93	70.31	68.93	68.53	39.22	67.74
Logistic Regression	66.57	66.50	66.62	66.50	66.48	33.12	66.37
AdaBoost	66.51	66.42	66.61	66.42	66.38	33.03	66.22
LDA	66.99	66.89	67.12	66.89	66.84	33.85	66.67
Decision Tree	83.67	83.62	83.82	83.62	83.64	67.44	83.55
MLP	67.65	67.46	68.46	67.46	67.13	35.90	66.48

**Table 6:** Performance metrics of the proposed method using ML models combined with CTGAN-generated synthetic data.

Model	Accuracy	AUC	Precision	Recall	F1-Score	MCC	G-Mean
Gradient Boosting	93.30%	93.26%	93.63%	93.26%	93.28%	86.90%	93.17%
Light Gradient Boosting Machine	93.11%	93.08%	93.48%	93.08%	93.09%	86.55%	92.97%
XGBoost	92.87%	92.84%	93.14%	92.84%	92.85%	85.97%	92.76%
Random Forest	92.56%	92.53%	92.82%	92.53%	92.54%	85.35%	92.45%
Tab Net	92.53%	92.52%	93.07%	92.52%	92.51%	85.58%	92.35%
Logistic Regression	91.51%	91.46%	92.48%	91.46%	91.46%	83.93%	91.15%
AdaBoost	90.16%	90.14%	90.34%	90.14%	90.14%	80.47%	90.08%
LDA	89.91%	89.84%	91.61%	89.84%	89.80%	81.44%	89.28%
Decision Tree	87.76%	87.77%	87.77%	87.77%	87.76%	75.54%	87.76%
**MLP**	**93.91%**	**93.87%**	**94.48%**	**93.87%**	**93.89%**	**88.34%**	**93.71%**

These findings indicate that CTGAN-based augmentation may provide advantages beyond simple resampling, particularly by generating samples that better reflect the variability observed in the original data^29,32^. This could help models such as MLP capture more nuanced feature interactions. However, further validation on external datasets is needed to determine whether these improvements generalize beyond the present cohort.

### Real vs. Synthetic data distribution of proposed method

To evaluate the fidelity of synthetic data generated by CTGAN-MLP, we compared the statistical properties of real and synthetic samples. Numerical features were assessed using histograms and Kernel Density Estimation (KDE), while categorical variables were examined through bar plots^39^.

**Fig. 3:**
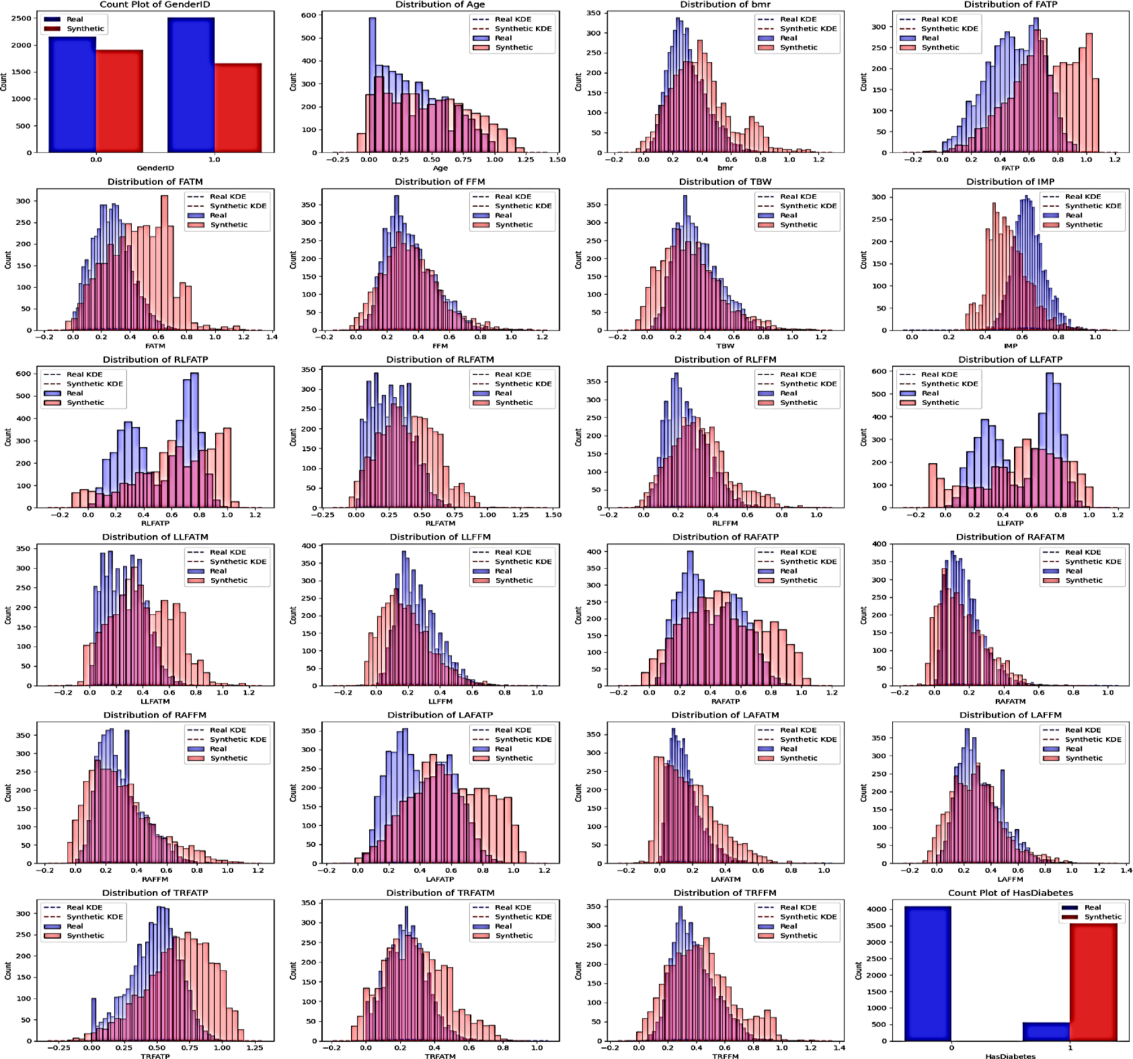
Comparison of real and synthetic data distributions generated by the CTGAN-MLP model, showing close alignment between both datasets.

As shown in Fig. 3, the synthetic data preserved the distributional characteristics of the original dataset across both feature types. This confirms that the CTGAN-MLP approach is effective in producing realistic synthetic samples that closely resemble true population distributions, thereby enhancing model robustness.

Based on Fig. 3, statistical comparison between real and CTGAN-generated data using the Kolmogorov–Smirnov test revealed no significant differences (*p* > 0.05) across key continuous features, confirming that the synthetic samples are statistically indistinguishable from the original data distribution. This high-fidelity replication suggests that CTGAN not only reproduces the marginal distributions but also maintains the joint relationships among correlated variables, such as fat percentage, fat-free mass, and basal metabolic rate. These results affirm that the synthetic data preserve physiologically plausible variability consistent with population-based body composition studies^22,30,39^.

### SHAP-Based feature importance of the proposed MLP

**Fig. 4:**
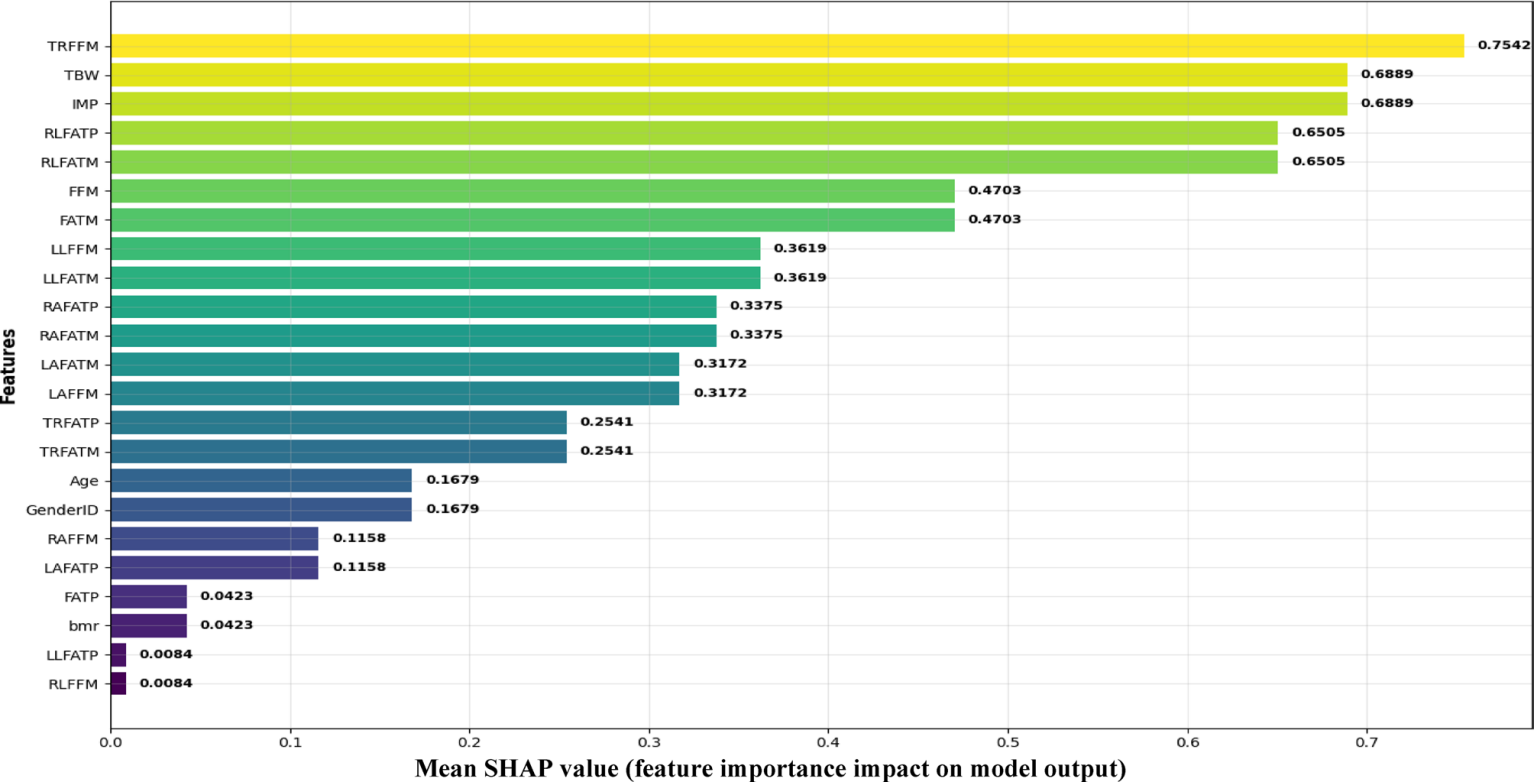
SHAP summary plot for the MLP model, showing how each body composition feature contributes to predicting diabetes.

To enhance interpretability, SHAP values were employed to analyze feature contributions within the MLP-based diabetes prediction model. The analysis revealed that body composition measures such as fat percentage, fat-free mass, and basal metabolic rate played the most influential roles in predicting diabetes status. SHAP values also highlighted the localized importance of segmental fat distribution, reinforcing the clinical relevance of regional adiposity patterns in diabetes risk. This analysis not only enhances transparency but also facilitates a deeper understanding of the physiological markers that drive predictions. As shown in Fig. 4, the SHAP summary plot for the MLP model illustrates the influence of individual body composition features on diabetes prediction.

The top SHAP features for CTGAN-MLP, total fat percentage, fat-free mass, and basal metabolic rate, are all known physiological measures of insulin resistance, metabolic efficiency, and energy expenditure, respectively, meaning that the model is identifying clinically meaningful markers that reflect known metabolic mechanisms^3,4,14^. The SHAP analysis therefore connects computational interpretability with biological relevance, suggesting that the model predictions are based on measurable physiological processes rather than statistical correlations.

### Mean SHAP value (feature importance impact on model output)

Following the SHAP analysis presented in Fig. 4, we further evaluated the robustness and consistency of the proposed CTGAN-MLP model using stratified 5-fold cross-validation. Table 7 summarizes the statistical performance of the model across the five folds, reporting the mean values, standard deviations, and 95% Confidence Interval (CI) for key metrics. Specifically, the ROC_AUC achieved an average of 92.43% with a standard deviation of 0.94% and a 95% confidence interval ranging from 91.60% to 93.26%. Similarly, Accuracy averaged 92.42% (Std = 0.95%, 95% CI = [91.59, 93.25]) and F1 Score reached 91.88% (Std = 1.10%, 95% CI = [90.91, 92.84]). These results indicate a high level of stability and reliability of the CTGAN-MLP model across different subsets of the dataset, confirming its strong generalization capability.

**Fig. 5:**
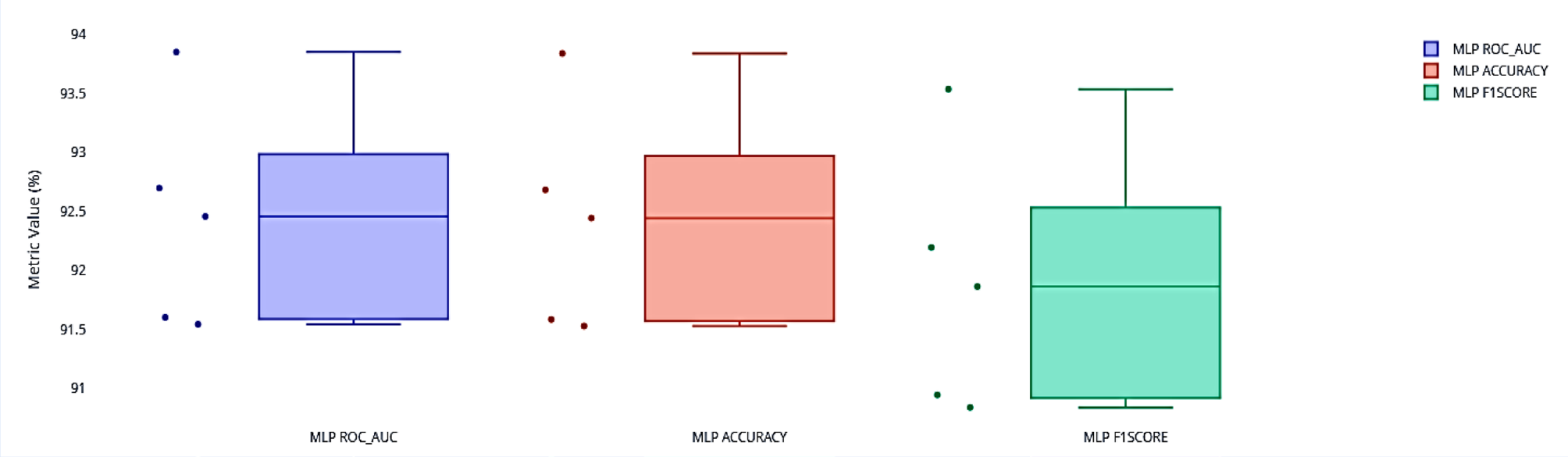
Distribution of CTGAN-MLP performance metrics across stratified 5-fold cross-validation. Evaluation metrics (AUC, Accuracy, and F1-Score)

The distribution of individual fold results is further illustrated in Fig. 5, which shows the spread of the ROC_AUC, Accuracy, and F1 Score across all five folds. The narrow distributions and absence of significant outliers reinforce the consistency of the model’s performance, suggesting that the augmentation with CTGAN effectively mitigates potential variability caused by class imbalance or limited sample sizes.

**Fig. 6:**
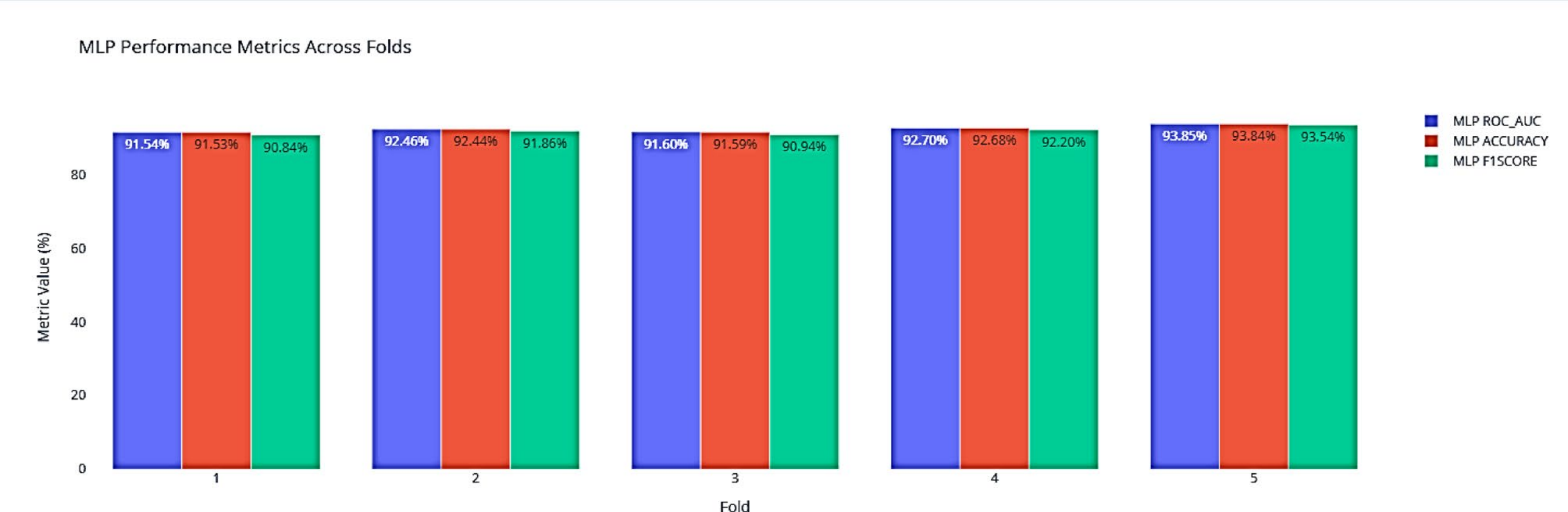
CTGAN-MLP performance metrics across individual folds.

Figure 6 complements this analysis by presenting a detailed view of the performance metrics for each fold. It highlights that the model maintains high accuracy and balanced predictive capability for both diabetic and non-diabetic classes across all folds. Together with the SHAP-based interpretability insights from Fig. 4, these results provide a comprehensive understanding of both the predictive power and the stability of the CTGAN-MLP framework, demonstrating its suitability for reliable diabetes risk prediction in real-world cohort data.

**Table 7:** Statistical summary of the proposed CTGAN-MLP model across stratified 5-Fold Cross-Validation.

Metrics	Mean (%)	Std (%)	95% CI (%)
AUC	92.43	0.94	[91.60, 93.26]
Accuracy	92.42	0.95	[91.59, 93.25]
F1 Score	91.88	1.10	[90.91, 92.84]

### Analysis on the fusion of CTGAN-MLP

The results presented in Tables 3, 4, 5 and 6 collectively highlight the superior effectiveness of the CTGAN-MLP configuration compared with traditional resampling techniques. While models trained on Random Under-Sampler, ADASYN, and SVM-SMOTE data achieved accuracies ranging from approximately 67% to 79%, incorporating CTGAN-generated synthetic samples led to a substantial improvement, with the MLP attaining 93.91% accuracy and an AUC of 93.87%. This improvement underscores the advantage of using a generative approach that preserves nonlinear feature dependencies rather than simply rebalancing class frequencies.

The enhanced performance of MLP can be explained by its ability to model complex, high-dimensional feature interactions in the CTGAN-augmented dataset. Through its nonlinear activation functions and multilayer structure, the MLP effectively captured subtle dependencies among interrelated physiological variables, such as trunk fat percentage, limb fat-free mass, and basal metabolic rate. In contrast, tree-based and linear classifiers, which rely on discrete or additive partitioning of the feature space, struggled to capture such continuous relationships. The CTGAN-generated data enriched the training space with realistic, diverse samples, helping the MLP generalize better to underrepresented metabolic patterns.

Furthermore, the integration of CTGAN and MLP can be interpreted as a complementary learning process. CTGAN expands the feature space by modeling latent interdependencies among body composition features, while the MLP disentangles these patterns to form distinct, clinically meaningful representations of diabetic and non-diabetic individuals. This synergy enhances both the discriminative power and physiological interpretability of the framework, as evidenced by the SHAP analysis (Fig. 4), which identified total fat percentage, regional fat distribution, and fat-free mass as the most influential predictors. Such features are known correlates of insulin resistance and glucose dysregulation^14,18,25^.

Despite these improvements, some limitations should be acknowledged. Generative models like CTGAN may replicate sampling biases present in the source data, particularly underrepresented subgroups with extreme body composition profiles. Moreover, the dataset used in this study originated from a single rural cohort in southern Iran^22,30^, which may restrict generalization across different ethnic or lifestyle populations. Future studies should therefore validate the CTGAN–MLP framework on larger, more heterogeneous datasets to confirm its robustness and ensure its applicability to broader demographic groups.

To provide a unified quantitative measure that integrates MLP clinical accuracy, CTGAN efficiency, and model stability, we provided the Augmented Medical Predictive Efficacy Score (AMPES), formulated as follows:

10 $$\begin{aligned}&\:{\mathrm{A}\mathrm{M}\mathrm{P}\mathrm{E}\mathrm{S}}_{\mathrm{M}\mathrm{L}\mathrm{P}-\mathrm{C}\mathrm{T}\mathrm{G}\mathrm{A}\mathrm{N}}=({w}_{a\:}.{Acc}_{MLP}^{aug}+{w}_{r}\:.\:{Recall}_{MLP}^{aug}+{w}_{u}\:.{AUROC}_{MLP}^{aug})\\&\times\:\frac{(1+\varDelta\:{Acc}_{MLP}+3.\:\varDelta\:{Recall}_{MLP})}{Ln(1+{\alpha\:}_{CTGAN})}\times\:(1-{D}_{JSD}^{CTGAN})\times\:\mathrm{e}\mathrm{x}\mathrm{p}(-\beta\:.\parallel{\nabla\:}_{{\theta\:}_{MLP}}{l}_{BCE}{\parallel}^{2})\end{aligned}$$

As shown in Table 8, each term of the AMPES formulation is described along with its intended role in summarizing different aspects of model behavior. The metric brings together elements related to predictive performance and characteristics of CTGAN-generated samples, offering a compact view of how generative augmentation and classifier training might jointly influence model outputs. However, its components should be interpreted as exploratory rather than definitive indicators of physiological or algorithmic mechanisms.

The AMPES formulation combines several complementary factors including accuracy- and recall-based indices, the relative contribution of CTGAN resampling, and measures of divergence between real and synthetic data to provide an aggregated score. These components are included to reflect potential influences of class balancing, sample diversity, and model optimization stability. Nonetheless, the weighting and functional form of the metric have not yet been validated across independent datasets, and further evaluation is required to determine its robustness.

Given these considerations, AMPES should be viewed as an initial attempt to summarize generative–predictive interactions within the CTGAN–MLP pipeline. While it may help highlight performance trends observed in this study, additional analyses on larger and more heterogeneous datasets are needed before it can be considered a reliable or broadly applicable metric for medical AI evaluation.

**Table 8:** Refined AMPES formulation integrating MLP predictive metrics and CTGAN generative balance for enhanced diabetes prediction accuracy.

Term	Represents	Why it belongs to MLP or CTGAN
$\:{Acc}_{MLP}^{aug}$, $\:{Recall}_{MLP}^{aug},{AUROC}_{MLP}^{aug}$	Final MLP predictive power after CTGAN augmentation	Pure MLP output on the mixed (real + synthetic) dataset
$\:\varDelta\:{Acc}_{MLP},\varDelta\:{Recall}_{MLP}$	Gain achieved only through CTGAN balancing	CTGAN contribution – improves recall by approximately 65–75%
$\:3.\:\varDelta\:{Recall}_{MLP}$	Triple weighting on recall gain	Clinical priority: missing a diabetic case is about 3× worse than a false alarm
$\:{\alpha\:}_{CTGAN}$	CTGAN oversampling ratio	CTGAN hyperparameter controlling the degree of data rebalancing
$\:Ln(1+{\alpha\:}_{CTGAN})$	Logarithmic penalty for excessive synthetic expansion	Prevents CTGAN from inflating performance by over-generation
$\:{D}_{JSD}^{CTGAN}$	Jensen–Shannon divergence between real and CTGAN-generated data	CTGAN quality gate; low values (≤ 0.09) ensure physiological plausibility
$\:\mathrm{e}\mathrm{x}\mathrm{p}(-\beta\:.\parallel{\nabla\:}_{{\theta\:}_{MLP}}{l}_{BCE}{\parallel}^{2})$	MLP gradient-stability term	MLP-specific bonus: smoother gradient landscape, less variance, higher final accuracy (β ≈ 0.5)

### 4.9. Comparison with State-of-the-Art

**Fig. 7:**
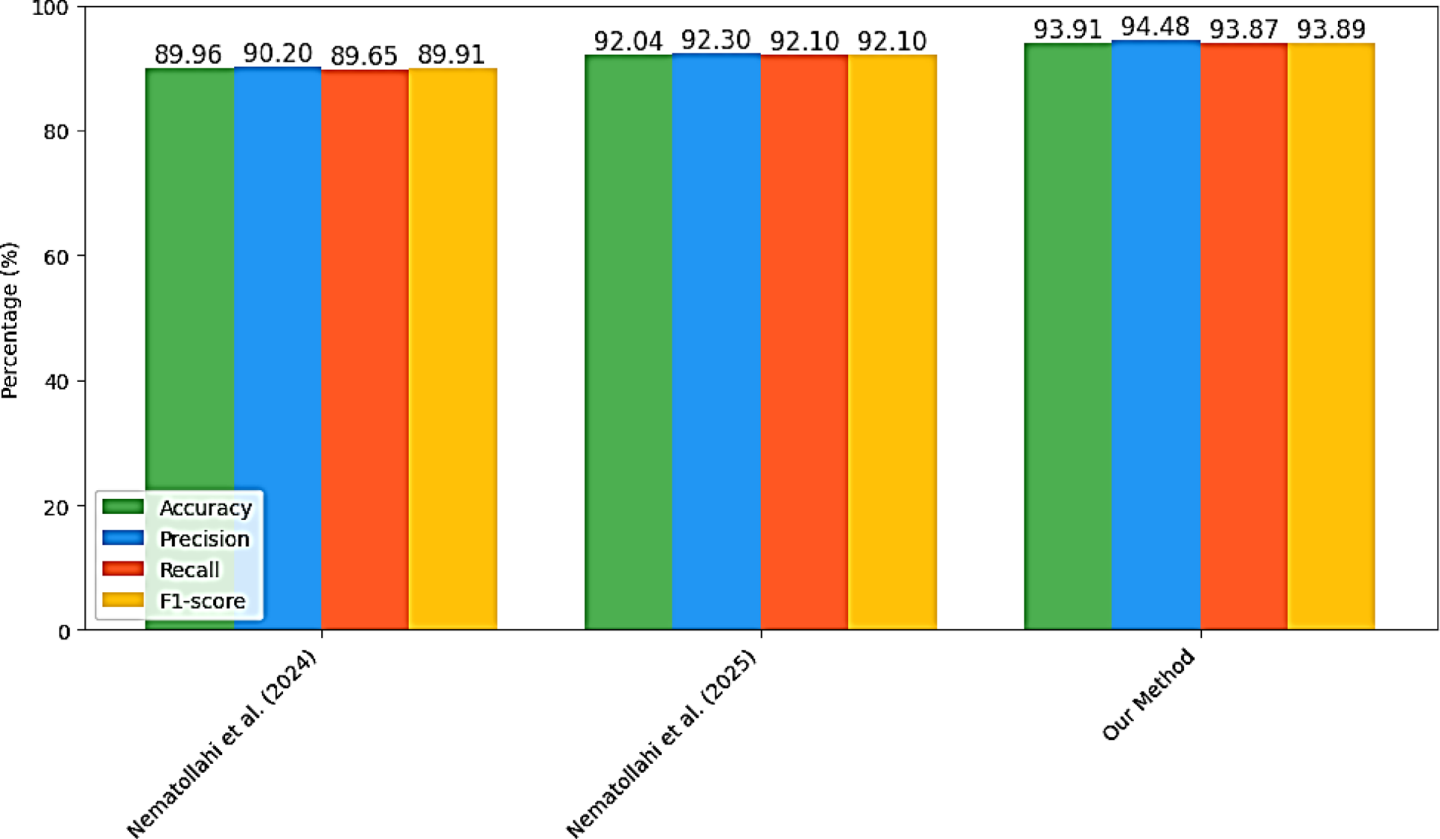
Performance comparison of the proposed method with state-of-the-art approaches in terms of Accuracy, Precision, Recall, and F1-score.

To evaluate the effectiveness of our proposed CTGAN-MLP framework, we compared it against previously published approaches using the same dataset of 4,661 participants. Table 9; Fig. 7 summarize this comparative analysis. Earlier studies by Nematollahi et al. reported accuracies of 89.96% and 92.04%, respectively, using XGBoost-based pipelines with feature selection and oversampling strategies^14,22^. In contrast, our method outperformed both, achieving 93.91% accuracy along with balanced improvements in precision, recall, and F1-score. These results highlight that combining CTGAN-based augmentation with MLP enhances predictive performance for diabetes prediction based on body composition data.

**Table 9:** Comparative performance of diabetes prediction methods on the same dataset (4,661 samples).

Study	Method	Accuracy (%)	Precision (%)	Recall (%)	F1-score (%)
Nematollahi et al., [22] (2024)	Feature Selection + XGBoost	89.96	90.20	89.65	89.91
Nematollahi et al., [14] (2025)	ANOVA + ADASYN + XGBoost	92.04	92.30	92.10	92.10
**Our Method**	**CTGAN + MLP**	**93.91**	**94.48**	**93.87**	**93.89**

These comparative results confirm that combining generative augmentation with nonlinear neural architectures enhances both numerical accuracy and physiological interpretability, a significant aspect that is often not emphasized in ensemble-based frameworks^19,20,24^. The CTGAN–MLP approach thus represents an important milestone in diabetes prediction research that has integrated machine learning performance with biological understanding to deliver a clinically interpretable and computationally efficient tool for early risk assessment.

## Limitations

Although the CTGAN–MLP framework achieved strong predictive performance, several methodological limitations should be critically acknowledged. While CTGAN helped mitigate class imbalance and generated statistically realistic samples, it may not fully capture the biological heterogeneity observed across broader populations. Subtle correlations specific to the Fasa Cohort could be learned and reinforced during generative training, resulting in cohort-dependent rather than population-generalizable representations. This suggests that part of the model’s performance reflects internal consistency within the cohort rather than true external generalization.

Moreover, the MLP’s high accuracy (93.91%) is closely tied to the structure of the CTGAN-generated feature space. If the generative process unintentionally synthesizes unrealistic feature combinations or latent correlations, the MLP may overfit to synthetic artifacts instead of physiologically meaningful patterns. This highlights a fundamental trade-off in generative augmentation: while increased diversity may improve recall, it can also reduce clinical fidelity when the synthetic distribution is imperfectly aligned with real data.

Although SHAP analysis enhanced interpretability by identifying influential predictors, it only partially reflects the complex nonlinear decision boundaries learned by the MLP. These boundaries cannot yet be fully mapped to underlying physiological mechanisms, limiting the immediate clinical interpretability of the framework. Additionally, the dataset represents a rural Iranian population whose socio-behavioral, environmental, and genetic characteristics may differ from urban or international cohorts, potentially constraining cross-population applicability.

In addition, this study relied solely on body-composition measurements without incorporating biochemical, lifestyle, or genetic variables known to influence diabetes risk. The absence of these covariates limits the ability of the model to represent the broader clinical context. Furthermore, the computational demands of CTGAN training and MLP hyperparameter tuning may restrict the practicality of deploying such systems in routine healthcare settings without adequate infrastructure.

These limitations collectively underscore the importance of external validation using larger and demographically diverse cohorts, the integration of biochemical and behavioral covariates, and the investigation of alternative generative approaches such as diffusion-based synthesizers that offer more explicit control over data realism and bias. Addressing these aspects will be essential to ensure methodological soundness and clinical translatability.

## Conclusion and future work

In this study, we developed a diabetes prediction framework that integrates body composition measurements with CTGAN-based data augmentation and evaluated its performance across multiple machine learning and deep learning models. The combination of CTGAN with an MLP classifier showed comparatively higher predictive performance in our experiments, suggesting that generative augmentation may help improve model robustness when dealing with class imbalance. However, these findings should be interpreted cautiously, as the improvements observed in this cohort may not fully generalize to other populations or clinical environments.

Although CTGAN allows the preservation of nonlinear dependencies within anthropometric features, reliance on synthesized data also carries the risk of replicating cohort-specific biases. Therefore, further validation is required before any strong conclusions about generalizability can be made. Future work will involve assessing this framework on larger and more diverse datasets such as the Tehran Lipid and Glucose Study and the Golestan Cohort Study—to evaluate its performance across different demographic and lifestyle contexts. Incorporating additional clinical variables, including biochemical and behavioral factors (e.g., HbA1c, fasting glucose, family history, and physical activity), may also enhance model interpretability and help provide more mechanistic rather than purely correlative insights.

We additionally plan to explore alternative generative approaches such as TVAE and diffusion-based models, which may offer more explicit control over synthetic data quality and reduce the risk of overfitting. Further analysis will examine the sensitivity of model performance to preprocessing strategies, imputation techniques, and feature-engineering choices. These steps will be essential for developing a reliable, interpretable, and ethically responsible predictive pipeline that can support personalized diabetes risk assessment and, ultimately, real-world clinical decision-making.

## Supplementary Information

Below is the link to the electronic supplementary material.

Supplementary Material 1

## Data Availability

The dataset analyzed in this study is a subset of the Fasa Cohort Study conducted in Iran. Due to privacy regulations and ethical considerations, the data cannot be publicly shared. However, researchers may request access to the dataset from the corresponding author, subject to appropriate ethical approvals and institutional agreements.

## Code availability

The preprocessing pipeline used in this study is publicly available on GitHub at:

https://github.com/MohammadMaftoun/Diabetes-Prediction-using-Body-Composition-Data.
