# Supplementary material for: Enhanced diabetes prediction using CTGAN-MLP approach on body composition data
Source: Sci Rep. 2025 Dec 10;16:2134. doi: 10.1038/s41598-025-31928-9 (PMC12808130; doi:10.1038/s41598-025-31928-9)
Supplement: Supplementary file 1 — Supplementary Material 1 [file 41598_2025_31928_MOESM1_ESM.docx]

**Figure S1.** Correlation Matrix of Body Composition Features

For a detailed view of the correlations among the body composition features, the correlation matrix is presented in Figure S1, accompanied by a heat map to illustrate the strength and direction of the relationships between features.


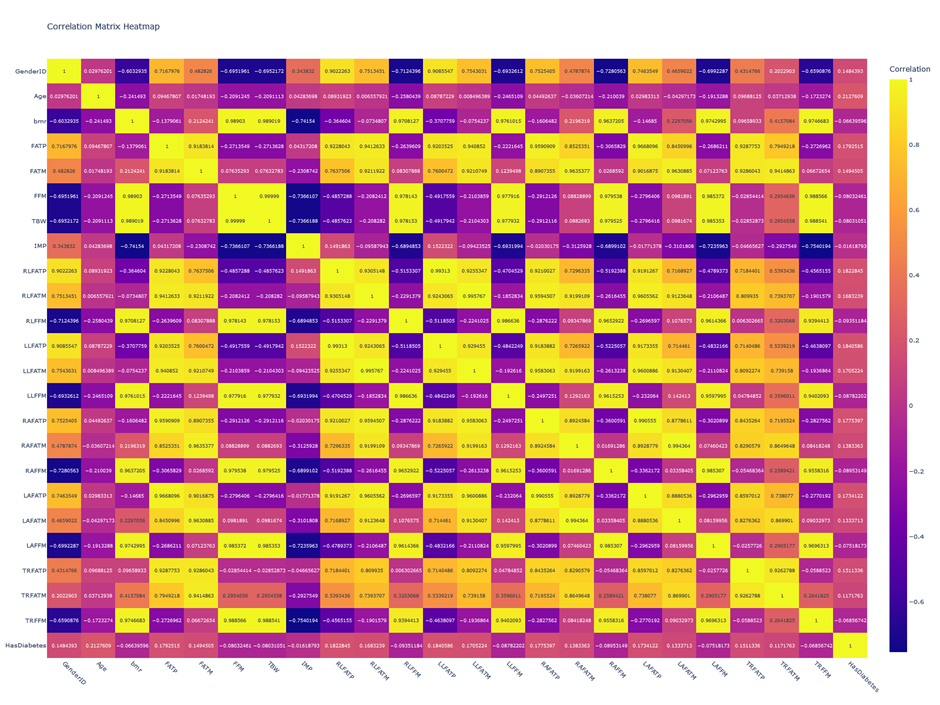


**Figure S1.** Correlation matrix of body composition features with heat map.
